# Rutin (quercetin-3-rutinoside) administered orally after experimental *Bothrops jararaca* envenomation prevents hemostatic, inflammatory and hemorrhagic disturbances

**DOI:** 10.1371/journal.pntd.0014786

**Published:** 2026-10-06

**Authors:** Natacha Ferreira de Oliveira, Adrielly Viveiros Torres, Marcelo Larami Santoro

**Affiliations:** 1 Laboratório de Pesquisa do Biotério Central, Instituto Butantan, São Paulo, Brazil; 2 Programa de Pós-graduação em Ciências Médicas, Faculdade de Medicina da Universidade de São Paulo, São Paulo, Brazil; Universidade Federal do Amazonas, BRAZIL

## Abstract

Bites caused by *Bothrops* snakes represent a major public health problem in South America and are associated with severe systemic and local clinical manifestations. *Bothrops* venom induces marked hemostatic and inflammatory disturbances that may result in hemorrhage, thrombocytopenia, tissue necrosis, and kidney injury. The recommended treatment for snakebite envenomation is intravenous administration of anti-*Bothrops* antivenom (SAB); however, this therapy has limited efficacy against venom-induced local tissue injury mediated by the release of inflammatory mediators. In this context, adjunct pharmacological strategies capable of delaying or attenuating envenomation effects before antivenom administration may be clinically valuable, particularly in remote settings. Rutin and its derivative, rutin succinate, are flavonoids with anti-inflammatory, antioxidant, and vasoprotective properties, and have been previously shown to mitigate venom-induced damage when administered parenterally prior to *Bothrops jararaca* (Bj) envenomation. Herein, we evaluated the efficacy of orally administered rutin, formulated for rapid gastrointestinal absorption (nano-rutin), used alone or in combination with SAB, in a murine model of Bj snakebite envenomation. Mice received nano-rutin by gavage 10 min after venom injection and were subsequently treated with or without SAB after 1 hour. Envenomed mice developed thrombocytopenia, leukocytosis, fibrinogen consumption, elevated levels of IL-6 and cell-free DNA (cfDNA), reduced DNAse activity, hypoalbuminemia, and marked local hemorrhage and dermonecrosis. Oral nano-rutin restored most evaluated systemic parameters, and significantly reduced local hemorrhage and dermonecrosis, even in the absence of SAB. These findings support nano-rutin as an effective complementary therapy to antivenom, particularly for mitigating local tissue damage induced by *Bothrops* venoms.

## Author summary

When people are bitten by *Bothrops* snakes, known in Brazil as “jararacas”, the venom causes serious health problems, including bleeding, blood clotting disturbances, and swelling and tissue damage at the site of the bite. The standard treatment is the administration of a specific antivenom produced in horses. However, for people living in remote areas, delays in reaching medical care are common, and antivenom alone is not very effective at reversing the local tissue damage caused by inflammation triggered by the venom. In this study, we explored whether a natural compound called rutin could help reduce the harmful effects of jararaca venom, especially when given in combination with the specific antivenom. Rutin is known for its anti-inflammatory, antioxidant, and blood vessel–protective properties. We tested whether rutin, when given orally, just 10 minutes after venom exposure, could lessen venom-induced damage, either on its own or in combination with antivenom. Our results showed that rutin helped prevent bleeding and tissue death at the venom injection site, reduced inflammation and helped restore blood clotting. Remarkably, these protective effects were observed even when rutin was used without antivenom. These findings suggest that rutin could be a valuable additional treatment for snakebite victims, particularly in situations where access to antivenom is delayed. This research opens new possibilities for supportive treatments, as oral administration of rutin, that help protect patients and reduce injury until full medical care is available.

## Introduction

In Brazil, *Bothrops* snakes are the primary cause of snakebite envenomation. According to data from the Ministry of Health, Brazil, 27,066 cases of snakebite envenomation were recorded in 2025, of which 19,911 were caused by *Bothrops* sp, resulting in 96 deaths [1]. In the Southeastern region, where São Paulo State is located, the species *Bothrops jararaca* is of paramount epidemiological and clinical importance, as it is the leading cause of recorded snakebite cases [2,3].

Patients envenomed by *Bothrops* snakes manifest both local and systemic symptoms. Local signs include pain, edema, bruising, and bleeding, which may progress to blistering and tissue necrosis. Systemic manifestations – which include coagulopathy, thrombocytopenia, bleeding, thrombotic microangiopathy, visceral and intracerebral hemorrhages, shock, myonecrosis, limb necrosis, acute kidney injury, compartment syndrome, and secondary infections – are responsible for increasing morbidity and, in some cases, leading to fatal outcomes [4–10]. These clinical manifestations are associated with the three key pathophysiological activities of *Bothrops* sp venoms: inflammatory, coagulant, and hemorrhagic [11].

*Bothrops* antivenom (SAB) has been the only treatment available and recommended by the Ministry of Health in Brazil for over a century [12], owing to its proven efficacy in primarily mitigating the systemic effects of envenomation. However, it is crucial to emphasize that managing local clinical manifestations remains a challenge in treating snakebite victims, as SAB, even when administered promptly, contributes minimally to resolving local tissue damage induced by *Bothrops* venoms, potentially failing to prevent severe sequelae, such as necrosis and limb amputation [13,14]. Beyond clinical limitations, global accessibility and production ethics present further hurdles. Currently, there is a critical shortage of antivenom supply in developing nations, particularly across Asia and Africa, due to logistical constraints and high production costs that severely limit access to this life-saving treatment [15]. Furthermore, conventional SAB production relies on horse immunization – a process that not only requires complex infrastructure, but that also raises increasing animal welfare concerns. These combined clinical, logistical and ethical hurdles underscore an urgent global demand for novel synthetic or natural therapeutic compounds.

Non-traditional therapeutic approaches, such as small-molecule inhibitors and plant-derived compounds, offer promising alternatives for immediate treatment by targeting enzymatic activity or chelating essential cofactors [16–18]. Varespladib, for example, is a small-molecule inhibitor that potently inhibits phospholipases A_2_, which are critical toxins responsible for paralysis, coagulotoxicity and systemic tissue damage [19,20]. Furthermore, natural flavonoids have demonstrated efficacy in inhibiting snake venom metalloproteinases and snake venom serine proteinases, countering neurotoxicity, and effectively reducing both tissue necrosis and oxidative stress in vital organs [16–18,21].

Flavonoids are bioactive naturally derived products with pharmacological potential [22]. In light of this, previous studies by our group demonstrated that the flavonoids rutin (quercetin-3-rutinoside) and rutin succinate, when administered parenterally prior to experimental envenomation, mitigate the production of reactive species, decrease bleeding time, delay fibrinogen consumption, markedly decrease local hemorrhage, and drastically reduce the lethal activity of *Bothrops jararaca* venom (Bjv) [23,24]. Thus, rutin emerged as a potential ancillary treatment for *Bothrops* snakebite envenomation [25]. When administered *per os*, rutin absorption is slow and dependent on metabolism by intestinal microflora [26,27]. To overcome these limitations, lipid-based nanoformulations (nano-rutin) can accelerate absorption, with peak levels observed within 1 h [28]. An optimized formulation combining triacetin (oil phase, stabilizing droplet formation), labrasol (primary surfactant, enhancing solubility and facilitating rutin absorption by increasing cell membrane permeability), kolliphor (RH 40) (co-surfactant for uniform dispersion of rutin into small droplets essential for bioavailability), and 1,2-propylene glycol (stabilizing co-surfactant) has been shown to enhance solubility and ensure rapid and efficient absorption of rutin [28].

Given the severe impact of *Bothrops* snake venoms on inflammation and hemostasis, particularly for those populations in remote areas – e.g., original communities in the Amazon – where antivenom access is delayed, new therapeutic alternatives are essential to complement SAB. Since previous studies have demonstrated the therapeutic potential of rutin to treat snakebite envenomation manifestations [23,24], the present study explored oral nano-rutin as a complementary therapeutic agent to SAB, when administered post Bjv administration. Using an animal model that simulates the clinical timeline of envenomation and subsequent treatment – i.e., a rescue experiment [29] –, nano-rutin was administered 10 min after venom injection to simulate an early first-aid intervention that could realistically be implemented immediately after snakebite in remote settings before patients had access to antivenom. In fact, oral nano-rutin formulations could be carried with patients, so that it could be ingested immediately after a snakebite accident. Thus, herein we investigated whether nano-rutin alone or in combination with SAB attenuates or prevents early systemic and local manifestations of envenomation. Our findings reveal that nano-rutin effectively neutralizes both local and systemic manifestations, especially when administered before SAB, thus providing a more accessible option for remote populations in which SAB is not promptly available.

## Methods

### Ethic statement

The experimental procedures with these animals were reviewed and approved by Institutional Animal Care and Use Committee in Instituto Butantan (Comissão de Ética no Uso de Animais do Instituto Butantan, CEUAIB, proc. 4491070319) and the Faculdade de Medicina, Universidade de São Paulo (1962/2023).

### *Bothrops jararaca* venom (Bjv), rutin and SAB

Bjv was obtained from the Laboratory of Herpetology, Instituto Butantan, as a pool of lyophilized venom from adult *B. jararaca* individuals (Sistema Nacional de Gestão do Patrimônio Genético e do Conhecimento Tradicional Associado, SisGen AF375C2). It was kept at -20°C. Rutin was obtained from Sigma (USA, code R5143). SAB was donated by the Instituto Butantan (lot 1305077).

### Animals

Male isogenic mice C57BL/6, weighing 30–35 g, were obtained from the Central Animal Facility at Instituto Butantan. Besides, all procedures involving the use of mice followed National Guidelines (Conselho Nacional de Controle de Experimentação Animal, CONCEA, Brazil), were in accordance with ARRIVE [30].

### *In vivo* protocol

To produce a model that presents clinical and laboratory signs of *Bothrops* snakebite envenomation in humans, Bjv was injected s.c. at the dorsal region of mice at a dose of 1.6 mg/kg b.w. [24]. This model induces a hemorrhagic area at the site of venom injection (dorsal area), abdominal wall hemorrhage, hemostatic disturbances, inflammation, but does not cause death (lethal dose 50% = 1.62 mg/kg, i.p.) [31]. Bjv was diluted immediately before use in sterile 0.85% NaCl solution (saline), at a concentration of 2 mg/mL, and injected s.c. at a volume of 4.0 mL/kg b.w. Rutin was administered p.o. at a dose of 70 mg/kg; it was diluted at a concentration of 20 mg/mL in a solution (vehicle) containing 10% glycerin triacetate (Sigma, code W200700), 30% labrasol (kindly donated by Gattefossé, Brazil), 30% kolliphor (RH 40) (Sigma, code 0776), and 30% (v/v) 1,2-propylene glycol 30% (Nuclear, code 311884, Brazil). The dose and dilution of rutin were established based on a previous study [28], in which this formulation (nano-rutin) showed a greater solubility and more rapid intestinal absorption by 1 h when administered orally. SAB was administered i.v. at the retro-orbital plexus at a dose of 0.32 mL/kg b.w, taking into consideration that 1mL of SAB neutralizes 5 mg of Bjv (product insert, Instituto Butantan).

Fig 1 depicts the experimental design. Mice received Bjv or saline (negative control) s.c. After 10 min, mice received saline (negative control), nano-rutin, or nano-rutin vehicle by gavage (100 μL) p.o. Nano-rutin was administered 10 min after venom injection to simulate an early first-aid intervention that could realistically be implemented immediately after snakebite in remote settings before access to antivenom. One hour after the administration of nano-rutin, mice received saline or SAB i.v. Antivenom administration 1 h after envenomation was chosen to reproduce a clinically relevant delay between snakebite and access to medical care, allowing the evaluation of nano-rutin as an adjunct treatment during this interval. At 6 or 12 h of Bjv administration –i.e., time points corresponding to the early phase of *Bothrops* envenomation [24,32] –, mice were anesthetized with isoflurane (induction and maintenance at 2.5%), and blood was collected by puncture of caudal vena cava into 1.0-mL plastic syringes and 24G needles. To reproduce the recovery observed in patients bitten by *B. jararaca* and treated with SAB, blood samples were collected from mice at 6 and 12 h after Bjv administration. These time intervals were selected based on the onset of recovery from hemostatic disturbances observed in envenomed patients, including normalization of whole-blood clotting time, recovery of platelet counts, and cessation of spontaneous bleeding, such as hematuria and gingival bleeding [13,33]. Blood was added to vials containing 3.2% trisodium citrate (1/10 volume) or Na_2_-EDTA (1 µL/1.0 mL of blood). SAB (1 volume) was added to 99 volumes of blood in all vials to prevent *in vitro* blood coagulation induced by Bjv. The blood anticoagulated with 3.2% sodium citrate was centrifuged at 1900 *g* for 10 min to obtain plasma, and samples were maintained at -80°C until processing. Blood anticoagulated with Na_2_-EDTA was used for complete blood cell counts (CBC). These analyses were performed using an automatic hematology counter BC-2800 Vet (Mindray, China). After blood collection, mice were euthanized by cervical dislocation, while still under anesthesia.

**Fig 1:**
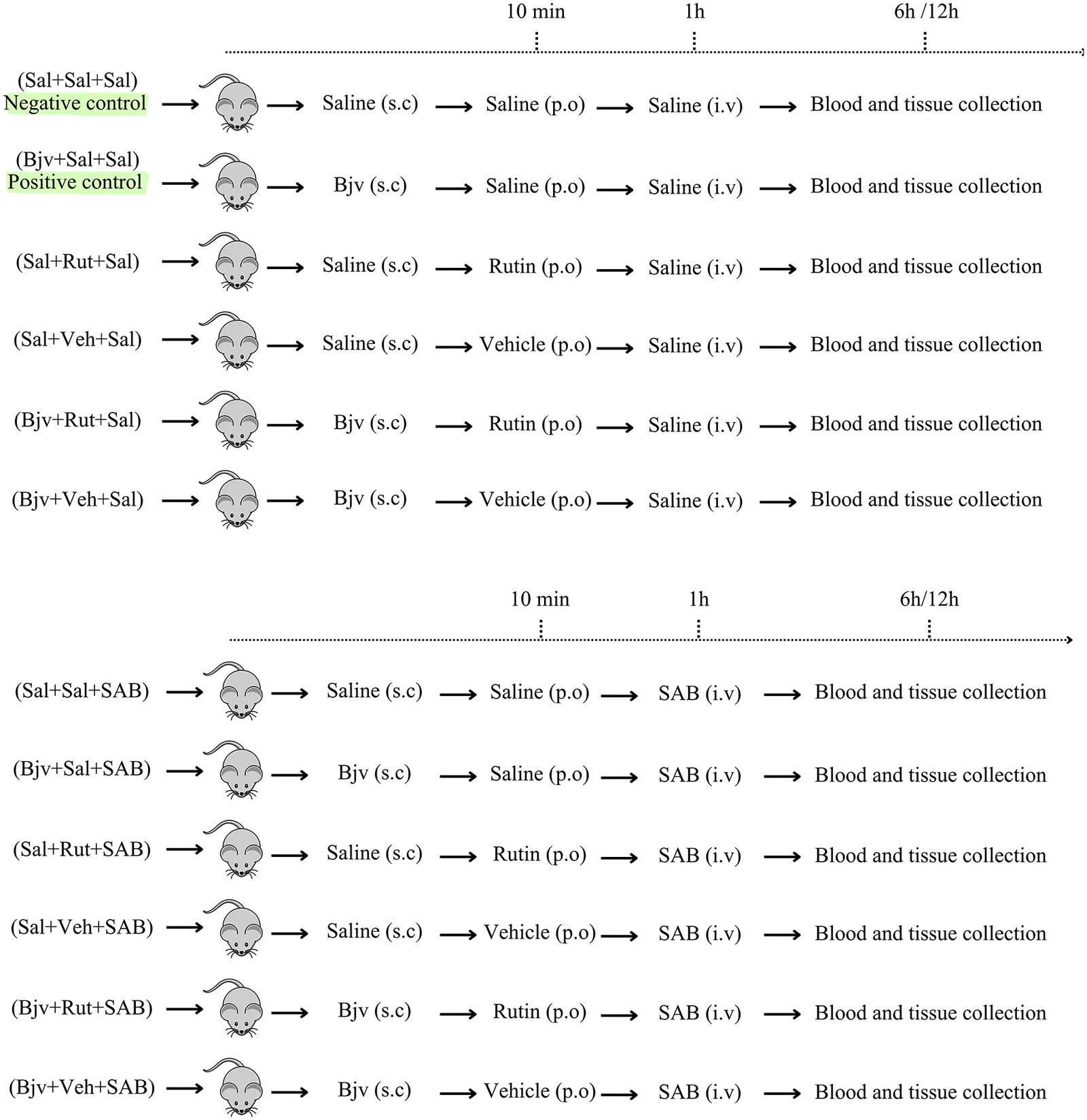
Schematic representation of experimental groups used for the study of the therapeutic effect of nano-rutin and/or SAB in experimental *B. jararaca* envenomation. C57BL/6 mice (30-35 g) were randomly allocated in experimental groups (n = 10/group).

### Fibrinogen assay

The fibrinogen assay was performed using citrated plasma following a modification of a previously described protocol [34]. Briefly, in 1.5-mL centrifugation tubes, 0.50 g of ground glass, 1 mL of saline, 25 μL of 10% tranexamic acid, 50 μL of plasma, and 35 μL of bovine thrombin (720 NIH-U) were added consecutively. The tubes were mixed and incubated in a water bath at 37°C for 15 min to form clots, followed by centrifugation at 1000 *g* for 5 min. After centrifugation, the clot was compressed, and the supernatant discarded. Saline (1 mL) was added, and the washing procedure was repeated twice, with the supernatant discarded each time. Finally, 1 mL of 10% NaOH was added to the tubes, and then they were sealed and heated at 100°C in a dry water bath for 10 min. A blank was prepared by adding 1 mL of saline, 25 μL of 10% tranexamic acid, 35 μL of bovine thrombin (720 NIH-U), and 1 mL of 10% NaOH to a separate 1.5-mL centrifugation tube. An aliquot (150 µL) of each sample was pipetted into each well of a UV-transparent 96-well microplate (Greiner, USA), and the absorbance was measured using a microplate reader at wavelengths of 280 nm and 315 nm against the blank. The initial reading of each sample was determined by the difference in optical densities (OD) between 280 nm and 315 nm. To calculate the fibrinogen concentration in samples, the pathlength (i.e., the distance the light travels through the sample) for a 150-μL sample was calculated, and a correction factor was applied to a 1.0-cm pathlength. To calculate the final concentration of fibrinogen in mg/mL in samples, the corrected sample reading was multiplied by 15.87, the extinction coefficient of fibrinogen.

### Assays of total protein and albumin assay in plasma

The total amount of protein and albumin in plasma samples was determined as described previously [35]. Aliquots of plasma samples (5 µL for albumin assay and 4 µL for total protein assay), standards (8 g/dL bovine serum albumin (BSA) two-fold serially diluted in saline), or blanks were pipetted in triplicate into the wells of a flat-bottom 96-well plate. Subsequently, for albumin assay, 200 µL of bromocresol green solution (0.50 mg/mL bromocresol green, 65 mM sodium hydroxide, 2.7% (v/v) lactic acid, and 1.0% (v/v) Tween 80) was added to each well; for total protein assay, 200 µL of biuret reagent (1.5% CuSO_4_·5H_2_O, 6% sodium potassium tartrate.4H_2_O, 3% NaOH, 0.1% KI, and) were added to each well. After homogenization, the plates were incubated at room temperature for 30 min to allow the reaction to occur. The absorbance was then read using a microplate reader (Loccus LMR-96) at a wavelength of 630 nm, and 540 nm, respectively, for albumin and total protein assays. For the albumin/globulin ratio determination, the amount of total globulin was calculated by subtracting the values of albumin concentration from those of the total protein concentration. The albumin/globulin ratio was then obtained by dividing the albumin values by the globulin ones [35] (S1 Table).

### IL-6 assay

The quantification of plasma levels of the pro-inflammatory cytokine IL-6 was evaluated using the Mouse Uncoated ELISA Kit (Invitrogen, USA). The assay was conducted in triplicate using citrated plasma samples (diluted 1:5), and 96-well MaxiSorp microplates (Nunc), following the manufacturer’s instructions. Absorbance readings were performed using the Loccus LMR-96 equipment, and IL-6 concentration analysis was done using CurveExpert software (version 1.4). The results were expressed as pg/mL of IL-6.

### Nitric oxide assay

For nitric oxide assay in plasma samples, the kit Nitric Oxide Assay (BioVision) was used. The assay was conducted in triplicate using citrated plasma samples, following the manufacturer’s instructions.

### Hemorrhagic area

Local hemorrhage at the site of venom injection was analyzed on the dorsal skin of the same animals used for blood collection. Images of the subcutaneous dorsal area were captured using an ImageScanner III (GE Healthcare, USA) at 600 dpi quality and saved in TIFF format. The images were analyzed using Adobe Photoshop software (version CS6) as previously described [23,36] to evaluate both the intensity of the hemorrhage and the hemorrhagic area. The intensity of the hemorrhage was calculated based on RGB color system values. Local hemorrhage was expressed both as values of hemorrhagic area (mm²) and as arbitrary hemorrhagic units (HaU), calculated based on the area and intensity values of the hemorrhage.

### Necrosis development

To assess the recovery of local tissue injury and histopathological changes evoked by Bjv, a separate experiment was carried out, so that the therapeutic alleviation of necrosis development by nano-rutin and/or SAB were also assessed at 72 h. This time interval was selected because dermonecrosis is fully established at this stage, allowing reliable macroscopic and histopathological evaluation of lesion severity and tissue repair. Necrosis was quantified after the intradermal injection of a higher concentration of Bjv (100 µg) into the dorsal region of mice (4 mg/kg) [37]. Five experimental groups were utilized, and after 10 min of i.d. injection, mice received either saline or rutin p.o, and after 1 h saline or SAB i.v. (Fig 2). After 72 h, mice were anesthetized, and blood samples were collected, as well as the dorsal skin was removed. The necrotic area was measured using Adobe Photoshop software (version CS6), and the results were expressed in necrosis area (mm²). Fragments of the lesion area were immersed in 2% buffered paraformaldehyde solution, and then processed for routine histopathological analyses and hematoxylin-eosin staining. Plasma samples from these mice were used for cell-free DNA (cfDNA) determination (see below). Only one investigator (MLS) analyzed histological sections, and the calculation of necrotic area was carried out independently by NFO in Adobe Photoshop.

**Fig 2:**
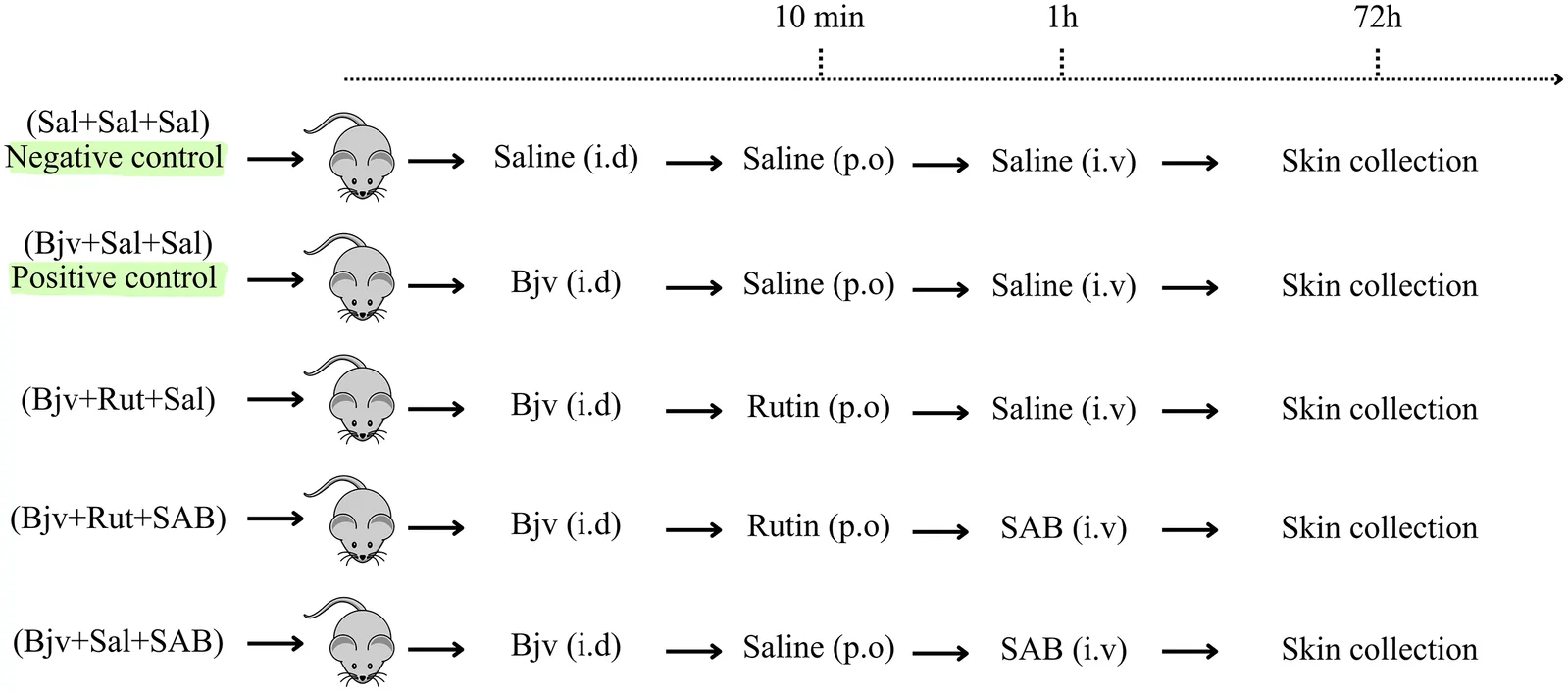
Schematic representation of experimental groups used for the study of the therapeutic effect of nano-rutin and/or SAB in skin necrosis induced by *B. jararaca* venom. C57BL/6 mice (30-35 g) were randomly allocated in experimental groups (n = 10/group).

### Cell-free DNA (cfDNA) in plasma samples

The quantification of cfDNA was performed using the Qubit dsDNA BR Assay Kit (Thermo Fisher Scientific, cat. no. Q33260). Initially, the working solution was prepared by diluting the dsDNA HS reagent 1:200 in dsDNA HS buffer. For the standards, 190 µL of the working solution and 10 µL of standard were added to an Eppendorf tube. For sample analysis, 180 µL of the working solution and 20 µL of plasma samples from mice used for necrosis development were added to 0.5-mL centrifugation tubes. The tubes were vortexed for 5 seconds and incubated at room temperature for 2 min, followed by measurement of standards and samples using a Qubit fluorometer (v. 2.0, Invitrogen, USA).

### Deoxyribonuclease (DNAse) assay

The DNase activity assay in plasma samples was performed using the DNA–methyl green reagent (Sigma-Aldrich, code D2376), by a modification of a described protocol [38]. Briefly, the DNA–methyl green solution was prepared by dissolving 20 mg of the reagent in 100 mL of buffer A (50 mM Tris, 7.5 mM MgCl_2_, pH 7.5). The solution was stirred overnight, and subsequently incubated at 37 °C for a few hours prior to use to ensure complete solubilization. DNase I (Agilent, code 400711–23, lot 0006123759) was prepared by reconstituting one vial containing the lyophilized enzyme (2600 U) in 260 µL of buffer B (10 mM Tris-HCl, 10 mM CaCl_2_, 10 mM MgCl_2_, and 50% glycerol (v/v), pH 7.5) and stored at -20 °C. A working solution was prepared by diluting 5 µL of this stock solution in buffer A to a final volume of 3 mL. For the assay, 170 µL of the DNA–methyl green solution was added to each well of a microplate, followed by the addition of 30 µL of buffer A (blank), plasma samples, or two-fold serial dilutions of DNAse I (16.67 to 0.13 U/mL) to generate the standard curve. The plate was incubated at 37 °C for 10 minutes, and absorbance was measured at 630 nm using a microplate reader. To calculate DNAse activity in plasma samples, the mean absorbance of the blank was subtracted from the absorbance values of the standard curve and samples. DNase activity was determined by fitting the data to a Morgan–Mercer–Flodin (MMF) nonlinear regression model using CurveExpert software (version 1.4).

### Statistical analyses

The number of mice allocated for each experimental group was based on the variation of blood cell counts of C57BL/6 from previous studies [24,39]; the software G*Power was used to calculate the sample size [40], using a priori power of 95%, an α error of 0.05%, and an effect size of 0.4. Ten mice were randomly allocated to each of eight treatment groups using Microsoft Excel.

Normal distribution and homoscedasticity of results were analyzed using the software STATA, version 15. For statistical comparisons among groups, two-way ANOVA was used, followed by Bonferroni *post hoc* test. Results were considered statistically significant when p < 0.05. Data were expressed as mean ± standard error of mean (S.E.M.). The Pearson’s correlation coefficient between necrosis area and cfDNA levels and linear regression between these variables were analyzed taking into account data from Sal + Sal + Sal and experimental groups together. The correlation coefficients, linear regressions and ANOVA analyses were performed in SPSS (version 27).

## Results and discussion

As reported below, favorable clinical and laboratory outcomes were observed when nano-rutin was administered orally 10 min after venom administration, with these effects being further enhanced by the administration of SAB 1h later. These results are promising, indicating that nano-rutin may serve as an ancillary therapeutic agent that can delay the onset of clinical manifestations of *Bothrops* snakebites until SAB can be administered.

To demonstrate that nano-rutin was indeed effective under our experimental conditions, various control groups were included. Initial statistical analyses were performed to determine whether control groups (Sal + Sal + SAB, Sal + Rut + Sal, Sal + Rut + SAB, Sal + Veh + Sal, and Sal + Veh + SAB) differed from one another and whether they exhibited similar patterns of changes, or no changes, over time when compared with the vehicle-treated group (Sal + Sal + Sal) (P > 0.05). Most of the variables analyzed in the control groups showed no significant changes over time, with the exception for WBC, lymphocyte and monocyte counts. Consequently, these control groups were excluded from the main analysis, and only the Sal + Sal + Sal group was retained as a reference control.

### Systemic inflammatory changes induced by Bjv are reverted by nano-rutin and SAB

Because *Bothrops* snakebite envenomation is known to induce an acute inflammatory response, we evaluated total and differential leukocyte counts together with plasma IL-6 and nitric oxide (NO) levels to determine whether nano-rutin and/or SAB modulated the early inflammatory response following Bjv administration. There was an increase in WBC counts (Fig 3a) at 6h after Bjv administration, which tended to recover at 12h. This increase in WBC counts was mainly driven by an increase in granulocyte and monocyte counts, consistent with previous findings showing that *Bothrops* envenomation rapidly induces an inflammatory response [24,33]. Rutin has been shown to inhibit the β_2_ subunit of integrins [41], which is expressed on leukocytes and has been demonstrated to play a role in their mobilization to the injury site. This mechanism may explain the increase in of lymphocyte (Fig 3b), granulocyte (Fig 3c), and monocytes counts (S1 Fig) observed in the Sal + Rut + Sal group. In patients bitten by *B. jararaca*, a rapid resolution of leukocytosis and neutrophilia is typically observed within 24 h after SAB administration [33]. In our study, no significant differences in total or differential leukocyte counts were detected at 6 or 12 h after Bjv administration among the Bjv + Sal + Sal, Bjv + Rut + Sal, Bjv + Sal + SAB, and Bjv + Rut + SAB groups, indicating that, within this time frame, the beneficial effects of these treatments on leukocyte counts could not be clearly demonstrated. Nevertheless, given that rutin is an anti-inflammatory agent, its effects were observed at later time points (see below) [23,24].

**Fig 3:**
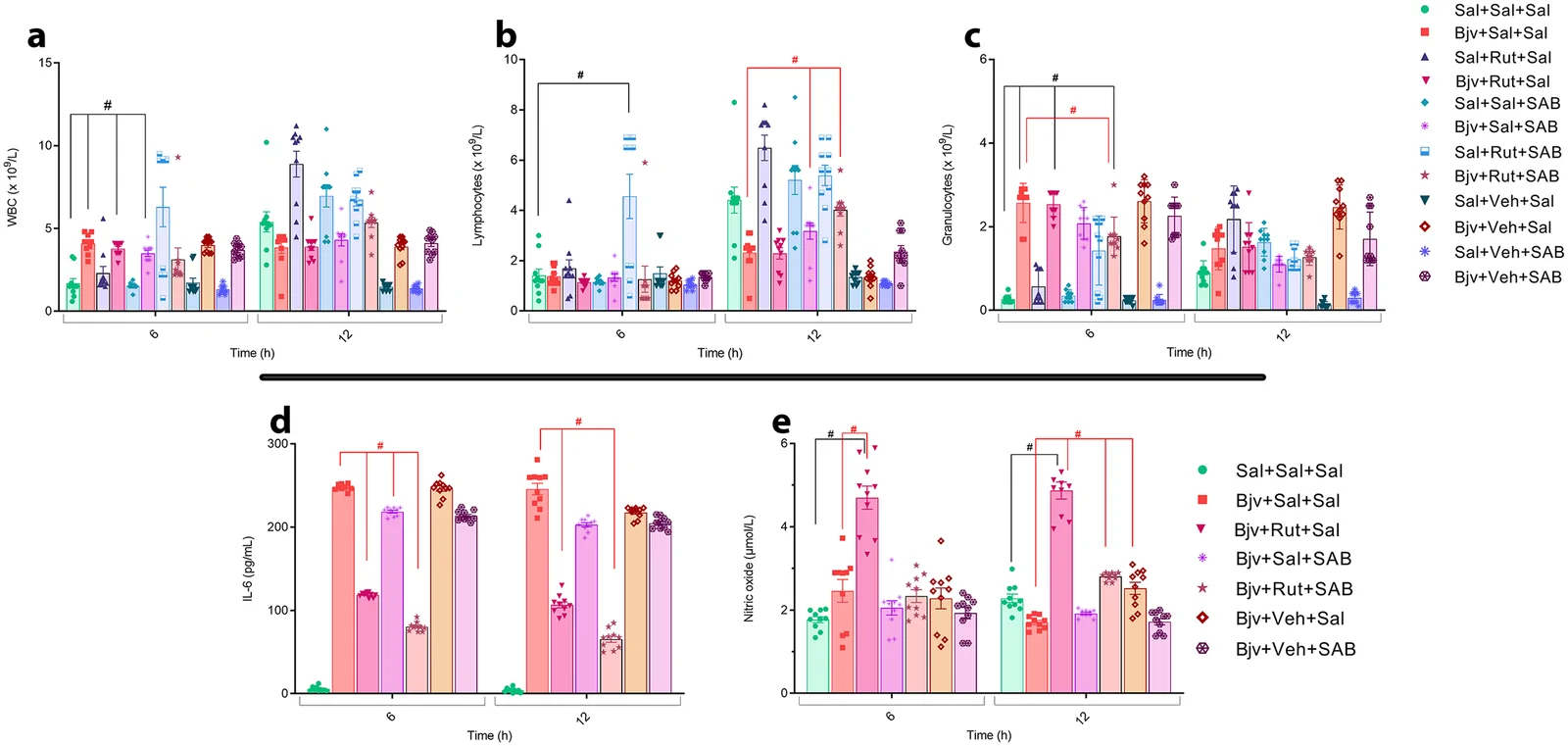
Inflammatory changes induced by Bjv, and their treatment with nano-rutin and/or SAB. Inflammation changes induced by Bjv and its treatment with nano-rutin and SAB parameters analyzed. Counts of circulating leukocytes (WBC, **a**), lymphocytes (**b**), granulocytes (**c**), and plasma levels of IL-6 (**d**) and nitric oxide (**e**) in mice 6 and 12 after administration of Bjv or saline, and treatment with nano-rutin (p.o., 10 min after Bjv administration), SAB (i.v. 1 h after Bjv administration) or both. Two-way ANOVA test was used, followed by the Bonferroni *post hoc* test. # p < 0.001 when compared to the negative control group (Sal + Sal + Sal) (black symbols and lines); # p < 0.001 when compared to the positive control group (Bjv + Sal + Sal) (red symbols and lines). Data are expressed as mean ± s.e.m. (n = 10/group).

Consistent with the observed changes in WBC counts, plasma IL-6 levels (Fig 3d) were significantly increased in all groups that received Bjv. Bjv triggers an acute inflammatory response mediated by the release of pro-inflammatory mediators, including IL-6, IL-1, IL-10, TNF-α, and interferon γ [42]. In contrast, nitric oxide (NO) levels in the Bjv + Sal + Sal group did not differ from those of the control group (Sal + Sal + Sal) at either 6 or 12 h (Fig 3e). These findings are in agreement with a previous report [42] showing that NO levels increase shortly (approximately at 2 h) after venom injection and subsequently decline over time. Interestingly, the Bjv + Rut + Sal group exhibited significantly higher NO levels compared with the other groups, indicating that rutin amplifies and prolongs Bjv-induced NO synthesis. Rutin has been shown to stimulate nitric oxide production in endothelial cells by increasing endothelial nitric oxide synthase (eNOS) protein expression [43]. In contrast, no significant increase in NO production was noted in the SAB-treated groups compared with the control, suggesting that SAB does not directly modulate NO synthesis.

Snake venom metalloproteinases (SVMPs) present in *Bothrops* venoms induce tissue damage and stimulate various cell types to synthetize IL-6 [44,45] and NO [46], thereby contributing to the local and systemic inflammatory responses characteristic of *B. jararaca* snakebite envenomation. Either SAB or nano-rutin administration diminished IL-6 levels, but nano-rutin was more effective than SAB in decreasing this cytokine. This finding suggests that although SAB is effective in neutralizing venom toxins, it has limited efficacy in controlling the Bjv-induced inflammatory response [14]. On the other hand, rutin and its nanoformulations – owing to their anti-inflammatory, antioxidant and vasoprotective properties [28,47–49] – may more effectively modulate BjV-induced inflammatory events. Its antioxidant activity further contributes to the attenuation of inflammation by reducing oxidative stress [23,24]. However, the most pronounced decrease in IL-6 levels was observed when both treatments were combined (Fig 3d), indicating that this therapeutic strategy was the most effective in limiting inflammation. These findings demonstrate that the use of both SAB and nano-rutin complements each other: while SAB neutralizes BjV, rutin mitigates the inflammatory response. This combined approach may therefore be particularly important for improving clinical outcomes following snakebite envenomation.

### Hemostatic changes: Fibrinogen levels recover earlier in mice treated with nano-rutin and SAB

Another hallmark of *B. jararaca* snakebite envenomation is the development of hemostatic disturbances. Clinically, they are manifested by several types of local and systemic bleeding, coagulopathy, hypofibrinogenemia, thrombocytopenia and platelet dysfunction, and secondary hyperfibrinolysis [50].

Regarding platelet counts (Fig 4a), a downward trend was observed in animals exposed to venom alone (Bjv + Sal + Sal). Interestingly, as previously observed for rutin administered alone via the intraperitoneal route [24], platelet counts were not improved by nano-rutin administration in comparison with the positive control group (Bjv + Sal + Sal). Conversely, groups treated with SAB showed improved recovery of platelet counts (p < 0.05); this recovery was more pronounced when nano-rutin and SAB were administered sequentially, suggesting that this combined treatment exerts a protective effect that may attenuate the severity of thrombocytopenia following the snakebite envenomation.

**Fig 4:**
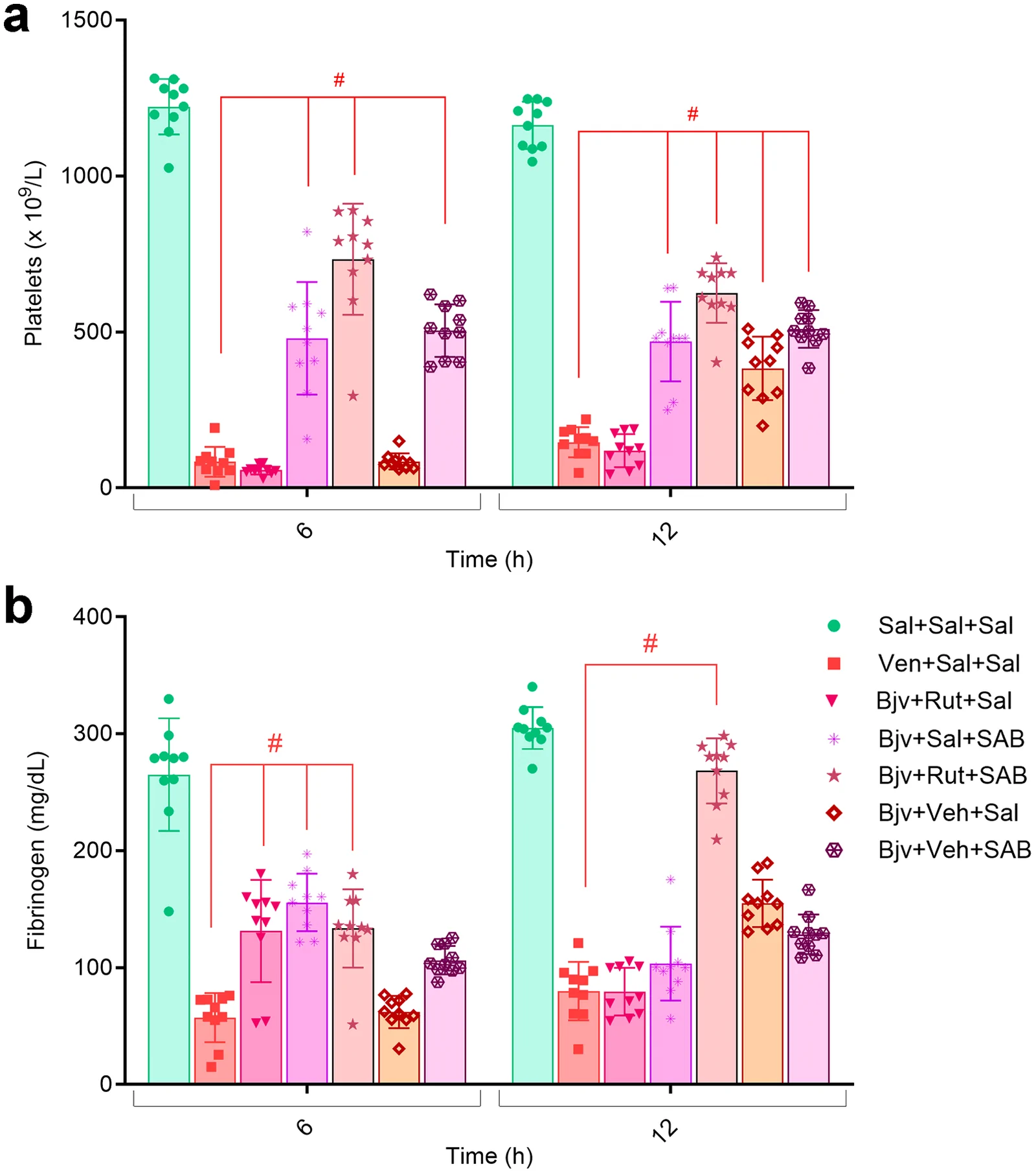
Hemostatic changes induced by Bjv and their treatment with nano-rutin and/or SAB. (**a**) platelet counts and (**b**) fibrinogen levels in mice 6 and 12 after administration of Bjv or saline, and treatment with nano-rutin (p.o., 10 min after Bjv administration), SAB (i.v. 1 h after Bjv administration) or both. Two-way ANOVA test was used, followed by the Bonferroni *post hoc* test. # p < 0.001 when compared to the negative control group (Sal + Sal + Sal) (black symbols and lines); # p < 0.001 when compared to the positive control group (Bjv + Sal + Sal) (red symbols and lines). Data are expressed as mean ± s.e.m. (n = 10/group).

As expected, mice treated with Bjv alone (Bjv + Sal + Sal) exhibited a marked reduction in fibrinogen levels at 6 and 12 h (Fig 4b). These findings are consistent with previous studies demonstrating that toxins present in Bjv rapidly induce blood coagulation activation, leading to fibrinogen consumption [24,51]. Particularly at 6 h after BjV injection, mice treated with nano-rutin (Bjv + Rut + Sal), SAB (Bjv + Sal + SAB) or the combination of both (Bjv + Rut + SAB) exhibited less pronounced fibrinogen consumption compared with the Bjv + Sal + Sal group. Importantly, within the first 6 h, fibrinogen levels in mice treated with nano-rutin alone (Bjv + Rut + Sal) were comparable to those treated with SAB, suggesting that nano-rutin may contribute to the preservation of hemostatic function, particularly in situations in which SAB administration is delayed. At 12 h, the combined treatment (Bjv + Rut + SAB) was more effective in restoring fibrinogen levels than SAB alone (Bjv + Sal + SAB), indicating that the association of nano-rutin and SAB is more effective in promoting fibrinogen recovery than either treatment administered individually.

### Nano-rutin reduces hemorrhage at the site of Bjv injection

Local hemorrhagic manifestations are a characteristic feature of *B. jararaca* snakebite envenomation [13]. In our experimental setting, Control groups exhibited minimal hemorrhagic areas at both 6 and 12 h, indicating that the subcutaneous injection procedure itself did not cause detectable local vascular damage. On the other hand, groups that received Bjv alone (Bjv + Sal + Sal) developed extensive and intense hemorrhagic areas (Fig 5a and 5b), with the highest values compared at both time points (Fig 5a and 5b). The extent of local hemorrhage remained relatively stable within each group at 6 and 12h (Fig 5b). Local hemorrhage induced by *Bothrops* venoms has been attributed primarily to SVMPs, particularly those of the PIII class, which degrade components of the extracellular matrix and compromise vascular integrity [52]. Remarkably, oral administration of nano-rutin after Bjv injection (Bjv + Rut + Sal), significantly reduced the extent of hemorrhage compared with the Bjv + Sal + Sal group (p < 0.05), demonstrating a clear protective effect against venom-induced hemorrhagic damage [24]. In fact, Seba in 1949 had already demonstrated that rutin was effective to inhibit the hemorrhagic activity of *Bothrops* venoms [53]. Although antivenoms incubated *in vitro* with Bjv or *Bothrops asper* venom neutralize hemorrhagic activity [54,55], administration of SAB alone (Bjv + Sal + SAB) after Bjv injection resulted in only a modest reduction in hemorrhage intensity, which was less pronounced than that observed in the Bjv + Rut + Sal group and did not differ significantly from the Bjv + Sal + Sal group. These findings corroborate a previous report indicating that SAB exerts only a limited palliative effect on Bjv-induced hemorrhage *in vivo* [14]. Strikingly, the sequential administration of nano-rutin and SAB (Bjv + Rut + SAB) led to a marked reduction in Bjv-induced vascular damage, greater than that observed with either treatment, demonstrating that this therapeutic association is highly effective in preventing the development of local hemorrhage (Fig 5a and 5b). The combined administration of nano-rutin and SAB provided a robust protective effect against venom-induced vascular damage, comparable to that observed when rutin was preincubated with Bjv prior to injection in mice [23,24]. Together, these findings indicate that the combination of nano-rutin and SAB resulted in greater protection than either treatment alone to limit vascular injury, thereby enhancing the protection against Bjv-induced tissue damage, and thus promoting a more effective therapeutic response.

**Fig 5:**
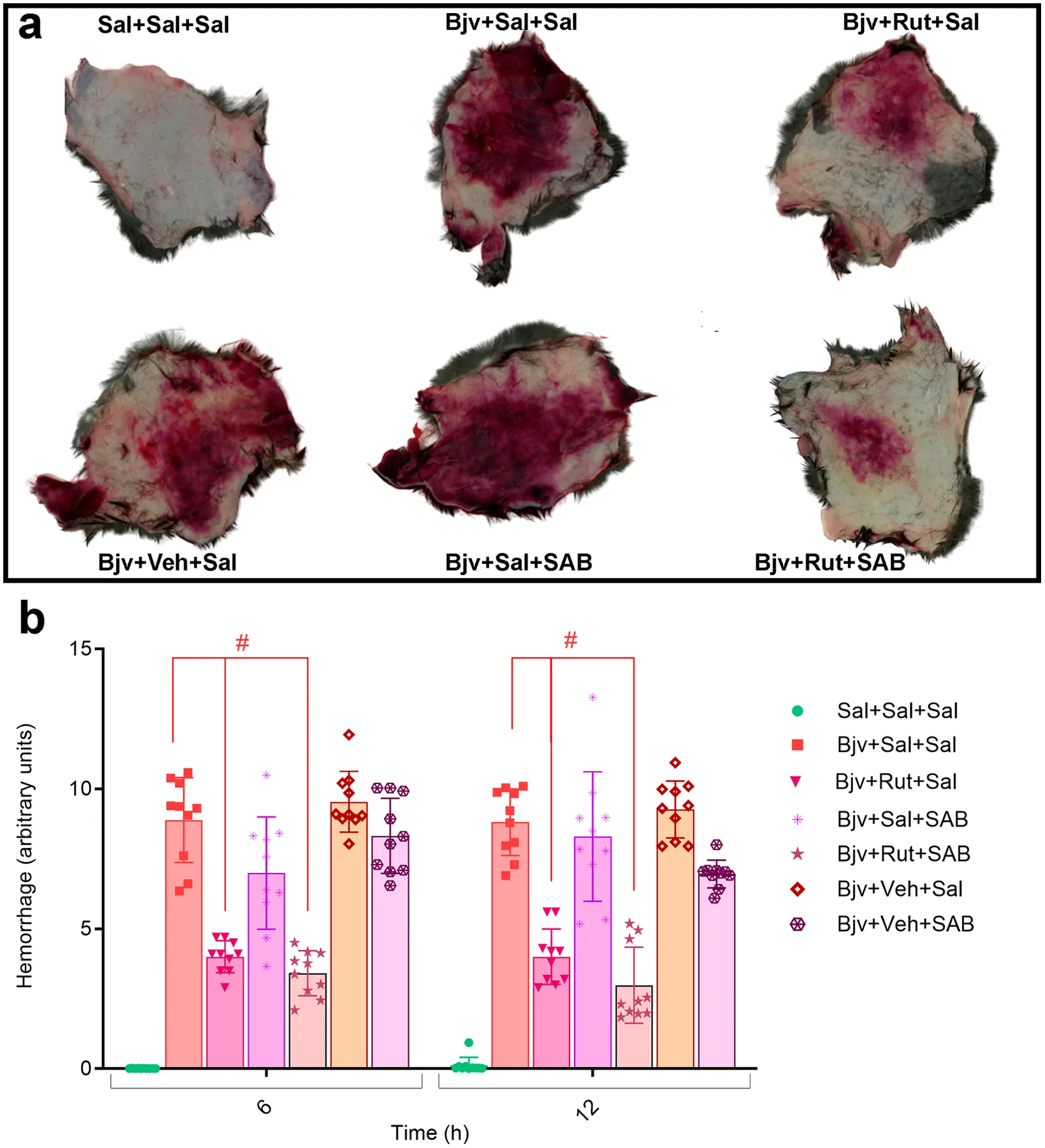
Local hemorrhage induced by Bjv, and its treatment with SAB and/or nano-rutin. (**a**) Representative images of local hemorrhage in experimental groups. (**b**) Intensity of local hemorrhage in mice 6 and 12 h. For comparison, two-way ANOVA test, followed by the Bonferroni *post hoc* test, was used. # p < 0.001 when compared to the positive control group (Bjv + Sal + Sal) (red symbols and lines). Data are expressed as mean ± s.e.m. (n = 10/group).

Bjv induces hemorrhage at the site of injection, as showed above, and systemic bleeding, both leading to a decrease in erythron parameters [24,33]. Consistent with these hemorrhagic effects, a significant reduction in RBC counts and hematocrit values (Fig 6a and 6b) was observed in mice that received Bjv alone (Bjv + Sal + Sal). This decrease became more pronounced over time, mirroring the clinical course observed in patients bitten by *B. jararaca* [33], and in experimental rodent models [24,56]. At 6 h, mice treated with either nano-rutin (Bjv + Rut + Sal) or SAB (Bjv + Sal + SAB) showed similar falls in RBC counts and hematocrit values, comparable to those observed in the Bjv + Sal + Sal group. However, the combined treatment with nano-rutin and SAB (Bjv + Rut + SAB) attenuated this decline. At 12 h, nano-rutin alone was no longer able to prevent the fall in RBC counts, whereas SAB partially mitigated this effect. Notably, the combined administration of nano-rutin and SAB improved the recovery of RBC counts. Together, these results suggest that the association of nano-rutin with SAB represents a promising strategy for improving the management of hematological disturbances and bleeding that occur during *Bothrops* snakebite envenomation, offering enhanced protection against Bjv-mediated hematological effects.

**Fig 6:**
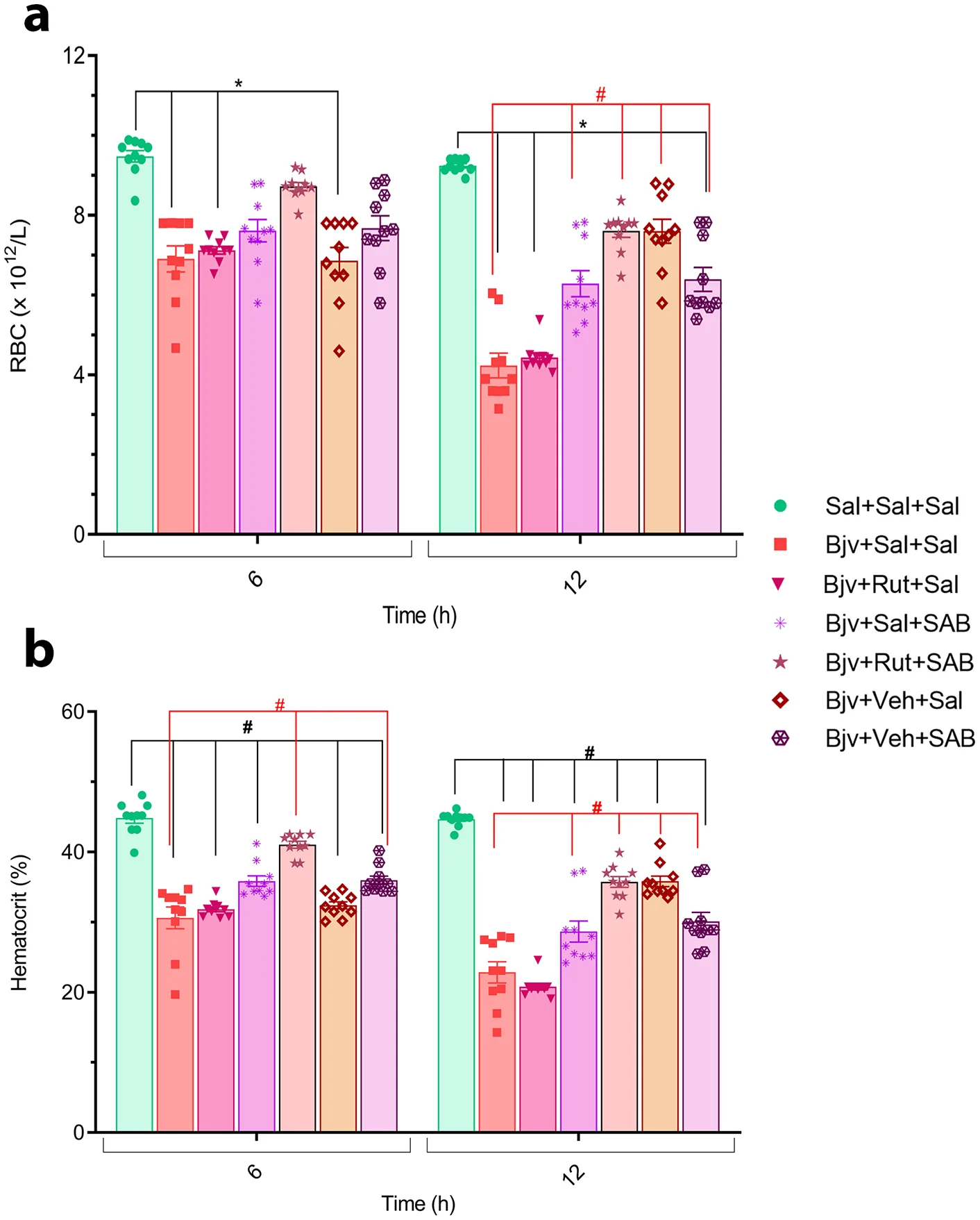
Values of red blood cell counts (RBC, a) and hematocrit (b) in mice 6 and 12 after administration of Bjv or saline, and treatment with nano-rutin (p.o., 10 min after Bjv administration), SAB (i.v. 1 h after Bjv administration) or both. Two-way ANOVA test was used, followed by the Bonferroni *post hoc* test. * p < 0.05, # p < 0.001 when compared to the negative control group (Sal + Sal + Sal) (black symbols and lines); # p < 0.001 when compared to the to the positive control group (Bjv + Sal + Sal) (red symbols and lines). Data are expressed as mean ± s.e.m. (n = 10/group).

### Both nano-rutin and SAB help maintain total protein and albumin levels after Bjv administration

Bjv induced hypoproteinemia and hypoalbuminemia in mice of the positive control group (Bjv + Sal + Sal) (Fig 7), similarly to what occurs in human patients bitten by *Bothrops* snakes [57]. Although local and systemic hemorrhage (Figs 5 and 6) could have contributed to decreases in total protein and albumin levels, BjV toxins and generated inflammatory mediators could also further increase vascular permeability and promote plasma protein extravasation systemically [58], a phenomenon that still needs scientific evidence for Bjv. The Sal + Sal + Sal group showed invariable total protein and albumin levels at both 6 and 12 h (Fig 7). Most Bjv-exposed groups exhibited a decrease in total protein levels at 12h, with the exception of the Bjv + Rut + Sal and Bjv + Rut + SAB groups (p < 0.001). The latter groups maintained relatively steady total protein levels over time, and the Bjv + Rut + SAB group even exhibited higher total protein values. On the other hand, albumin levels were already reduced in Bjv-treated mice at 6 h, particularly in the Bjv + Sal + Sal and Bjv + Veh + Sal groups (p < 0.05). Bjv-exposed groups treated with SAB preserved albumin levels more effectively than those treated with nano-rutin alone. At 12h, albumin levels remained reduced in all Bjv-treated groups, and only the association of nano-rutin and SAB restored albumin levels to values comparable to those of the control groups. These findings suggest that nano-rutin and SAB attenuates protein and albumin loss. Nano-rutin possibly preserves endothelial barrier by modulating pro-inflammatory mediators and stabilizing physiological inhibitors, and SAB by inhibiting Bjv toxins that alter endothelial function [43,59]. These findings indicate that Bjv and its associated inflammatory response increase vascular permeability, leading to protein extravasation, particularly of albumin, into the extravascular compartment.

**Fig 7:**
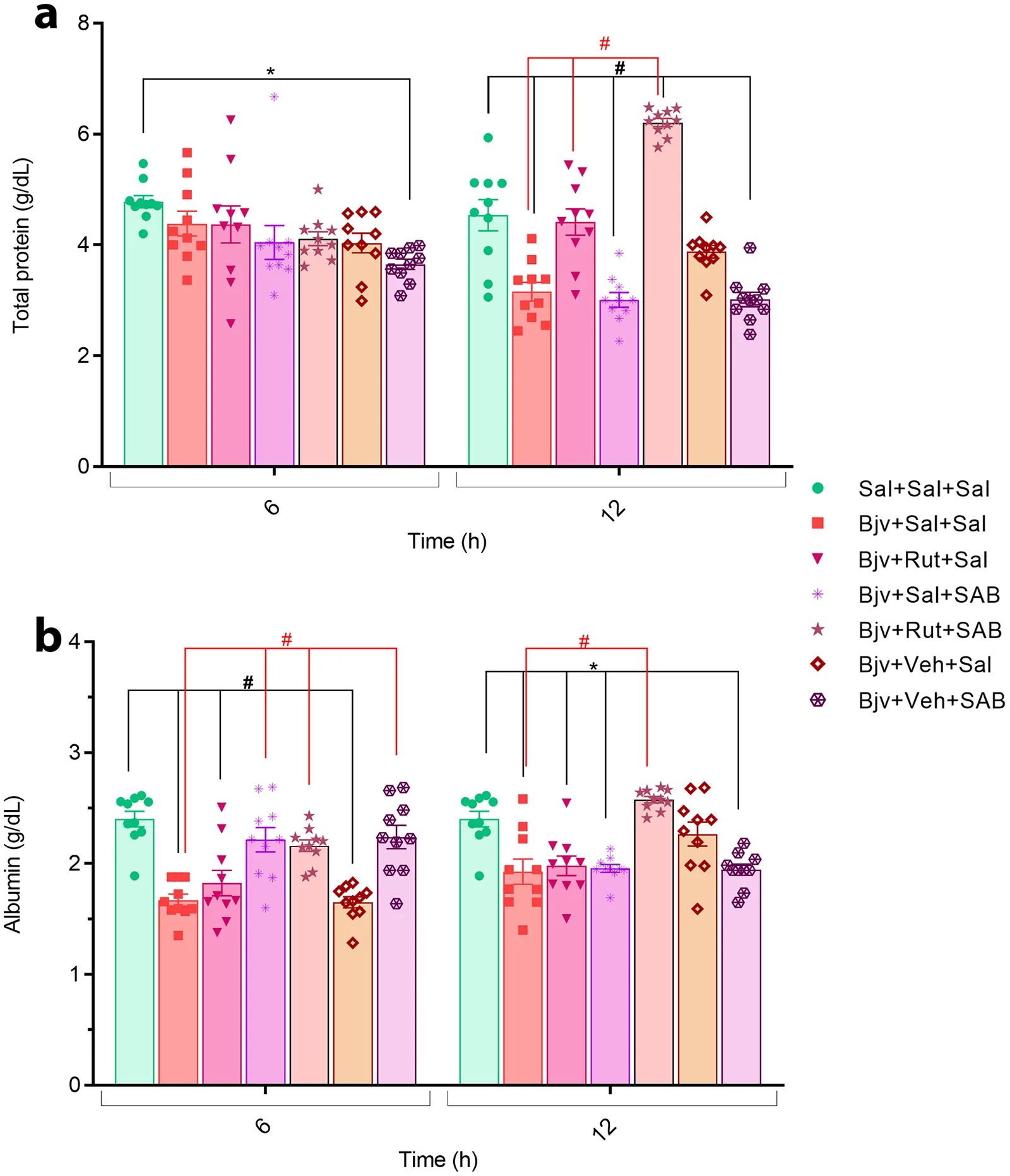
Plasma level of total protein (a) and albumin (b) in mice 6 and 12h after administration of treatments with saline, venom, nano-rutin, SAB or nano-rutin+SAB. The two-way ANOVA test was used, followed by the Bonferroni *post hoc* test. * p < 0.05, # p < 0.001 when compared to the negative control group (Sal + Sal + Sal) (black symbols and lines); # p < 0.001 when compared to the to the positive control group (Bjv + Sal + Sal) (red symbols and lines). Data are expressed as mean ± s.e.m. (n = 10/group).

### Nano-rutin reduces the development of Bjv-induced dermonecrosis

Local tissue necrosis is among the most severe complications of *Bothrops* snakebites, as it can lead to permanent disability or amputation, particularly for rural workers whose livelihoods depend on physical labor. In order to evaluate whether nano-rutin and/or SAB could reduce or prevent this adverse outcome, a high dose of Bjv known to induce local necrosis in mice was administered [37]. Macroscopic analysis revealed that treatment with nano-rutin or SAB significantly reduced the necrotic area compared with the BjV + Sal + Sal group (Fig 8a and 8b). Notably, the reduction in lesion size achieved with Bjv + Rut + Sal was comparable to that of nano-rutin combined with SAB. In contrast, Bjv + Sal + SAB demonstrated only a moderate effect, which was inferior to nano-rutin alone (Fig 8a and 8b). These findings demonstrated that nano-rutin administered after a high-dose injection of Bjv exerted a marked protective effect against the development of dermonecrosis.

**Fig 8:**
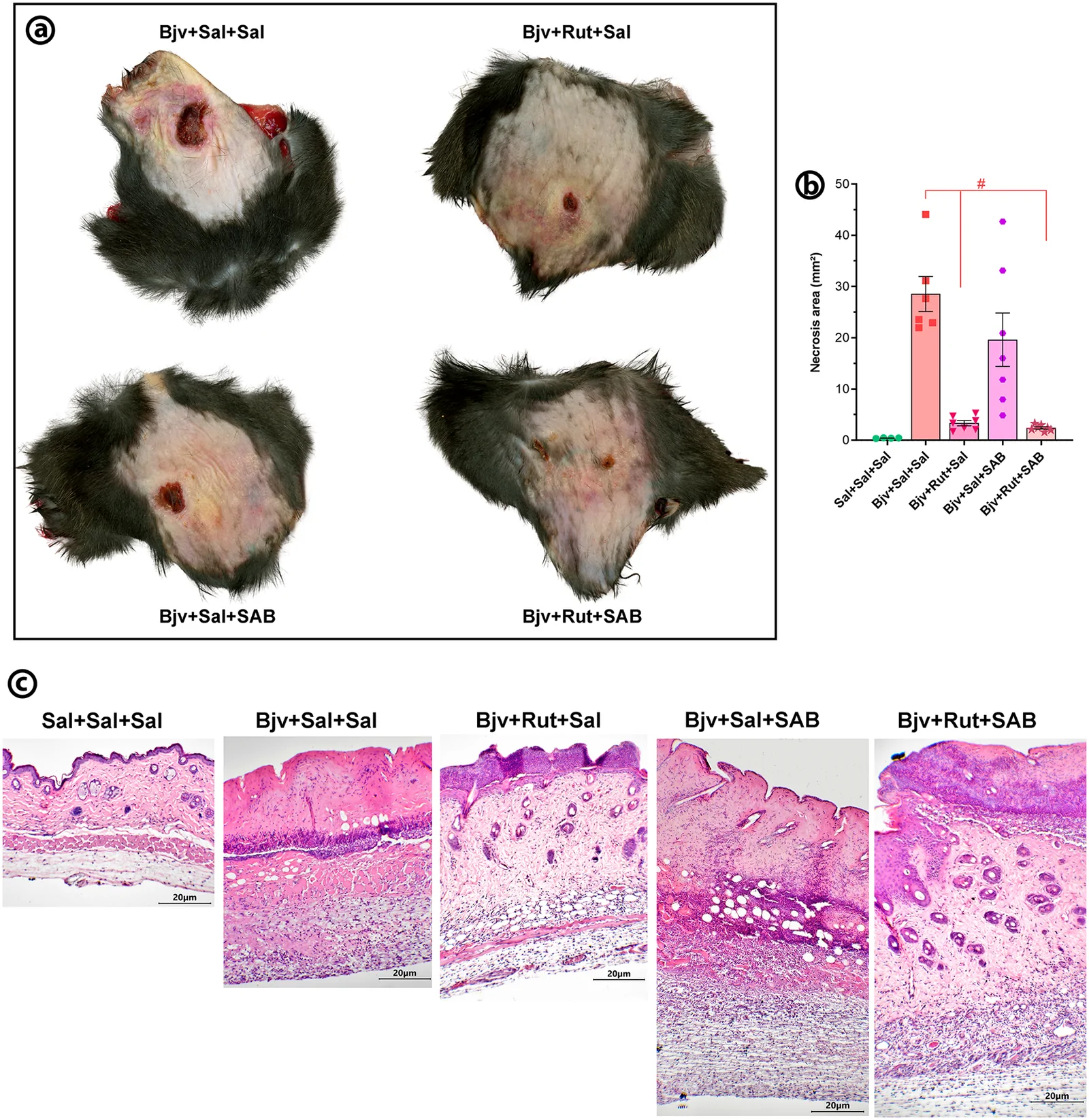
Necrosis 72h after i.d. administration of Bjv to mice, and treatments with nano-rutin (p.o) and/or SAB (i.v.) (see Fig 2). (**a**) Representative images of necrosis in each experimental group. (**b**) Area of necrosis. One-way ANOVA test was used, followed by the Bonferroni *post hoc* test. # p < 0.001 when compared to the positive control group (Bjv + Sal + Sal) (red symbols and lines). Data are expressed as mean ± s.e.m. (n = 7/group). (**c**) Representative H&E-stained sections of dorsal skin from mice three days after *Bothrops jararaca* venom (Bjv) inoculation. Control skin (Sal + Sal + Sal) shows preserved epidermal and dermal architecture. Bjv alone (Bjv + Sal + Sal) induces extensive epidermal loss, dermal necrosis, inflammatory infiltration, and destruction of adnexal structures. Nano-rutin treatment (Bjv + Rut + Sal) partially attenuates tissue damage, with preservation of epidermis and dermal organization. Antivenom alone (Bjv + Sal + SAB) fails to prevent severe dermal and muscular disorganization. Combined treatment (Bjv + Rut + SAB) reduces lesion severity, showing partial epidermal regeneration and improved dermal preservation. Scale bar = 20 µm. For details, see the text.

To have a global view of what was occurring at the site of venom injection, histological analysis of the dorsal skin of these mice was also performed 72 h, thereby allowing detailed evaluation of the wound-healing process (Fig 8c). Control mice injected only with saline (Sal + Sal + Sal group) exhibited normal skin architecture, including a thin epidermis; dermis containing collagen fibers, fibroblasts, sebaceous glands, hair follicles and few blood vessels; a relatively thin hypodermis composed of adipose tissue, the *panniculus carnosus* (striated skeletal muscle), and the adventitia (Fig 8c). Mice in the Bjv + Sal + Sal group (positive control for necrosis) exhibited an extensive necrotic area (Fig 8a), with an average area of approximately 30 mm² (Fig 8b), characterized by well-defined necrotic regions and evident tissue loss. Mice from Bjv + Sal + Sal group displayed evident necrotic tissue formation on the lesion surface, accompanied by thinning or complete loss of the epidermal layer. In the dermis, marked destruction of collagen fiber architecture and vacuole formation were observed, along with disorganization of the muscle cell layer. The adventitia showed plasma extravasation (interstitial fluid accumulation) and leukocyte infiltration, predominantly mononuclear cells. Hair follicles were destroyed and no longer detectable. (Fig 8c). These observations are consistent with previous studies describing the pronounced dermonecrosis activity of Bjv, which has been primarily ascribed to the combined actions of SVMP, IL-6 and TNF-α receptors [55,60–63]. They are also similar to that observed in patients bitten by *Bothrops atrox* [64]. These mechanisms promote extracellular matrix degradation, and intense local inflammatory responses.

In contrast, administration of nano-rutin (Bjv + Rut + Sal group) did not prevent the development of Bjv-induced cutaneous lesions, but attenuated their severity compared with the Bjv + Sal + Sal group (Fig 8c). Necrotic tissue was still present on the surface; however, the epidermal layer was preserved. The dermis and hair follicles remained largely intact, and collagen fiber architecture was less disrupted when compared to the Bjv + Sal + Sal group. The hypodermis was clearly identifiable and contained inflammatory cells. The cytoplasm of muscle fibers remained swelled with loss of normal striations. These findings suggest that nano-rutin preserved the collagen fiber architecture, likely due to its antioxidant protective effects, therefore preventing the spread of Bjv toxins and the further increment of the inflammatory reaction. On the other hand, mice treated with antivenom alone (Bjv + Sal + SAB group) exhibited thicker superficial necrotic tissue and complete epidermal disorganization (Fig 8c). The dermis contained thickened collagen fibers forming isolated aggregates. The hypodermis showed intense inflammatory infiltration extending into the muscle layer. Muscle fibers were disorganized, and their cytoplasm was swelled and lacked characteristic striations. These findings indicate that SAB alone was unable to prevent Bjv-induced tissue damage, with histological alterations resembling those observed in the positive control group (Bjv + Sal + Sal) (Fig 8c). Comparatively, greater preservation of epidermal and dermal structures was observed in nano-rutin-treated animals, whereas SAB treatment alone did not protect against epidermal and muscular disorganization. These results indicate that nano-rutin was more effective in preventing the development of dermonecrosis than SAB alone. Although SAB (Bjv + Sal + SAB) only partially limited necrotic lesion development (Fig 8a–8c), a clear cooperative effect occurred when both therapeutic agents were used together (Fig 8a–8c). Notably, mice receiving both nano-rutin and SAB (Bjv + Rut + SAB group) exhibited less severe Bjv-induced lesions. Although superficial necrotic tissue was still present, the epidermis adjacent to the lesion showed proliferative activity and initiation of dermal coverage. As observed in the Bjv + Rut + Sal group, dermal structures – including hair follicles and fibroblasts – were better preserved. The hypodermis, between the dermis and the *panniculus carnosus*, showed marked mononuclear cell infiltration and increased vascularization. The muscle layer remained partially disorganized, with inflammatory cells surrounding muscle fibers. The marked reduction in dermonecrosis observed in mice treated with nano-rutin and SAB suggests that these agents act at complementary stages of the pathological process.

Rutin, a flavonoid widely recognized for its antioxidant and anti-inflammatory properties, may exert its effects by scavenging reactive oxygen species, inhibiting the production of TNF-α, IL-1β, and IL-6, and reducing COX-2 expression during Bjv-induced tissue injury [47,49,65,66]. In turn, SAB acts primarily by neutralizing circulating toxic components of Bjv, showing limited efficacy in mitigating local inflammatory damage. [13,55]. In summary, the combined therapeutic use of nano-rutin and SAB enhances protection against Bjv-induced dermonecrosis, with nano-rutin attenuating local inflammatory and oxidative damage, and SAB reducing the systemic burden of venom toxins.

Blood samples collected from the mice used to assess dermonecrosis were also analyzed to determine whether the treatments modulated systemic inflammatory and hemostatic alterations 72 h after Bjv administration (Fig 9). Bjv administration resulted in increased WBC, granulocyte, and lymphocyte counts compared with the Sal + Sal + Sal group (Fig 9a–9c). Treatment with nano-rutin, SAB, or their combination reduced these inflammatory cell counts compared with Bjv + Sal + Sal, although values did not consistently return to those of the negative control group. The Bjv + Sal + Sal also had marked thrombocytopenia, increased mean platelet volume (MPV), and reduced fibrinogen levels (Fig 9d–9f). Nano-rutin and/or SAB partially improved these hemostatic alterations. Following Bjv administration, RBC counts decreased while RDW increased; these alterated hematological parameters were partially attenuated by the treatments, although none achieved complete restoration (Fig 9g and 9h).

**Fig 9:**
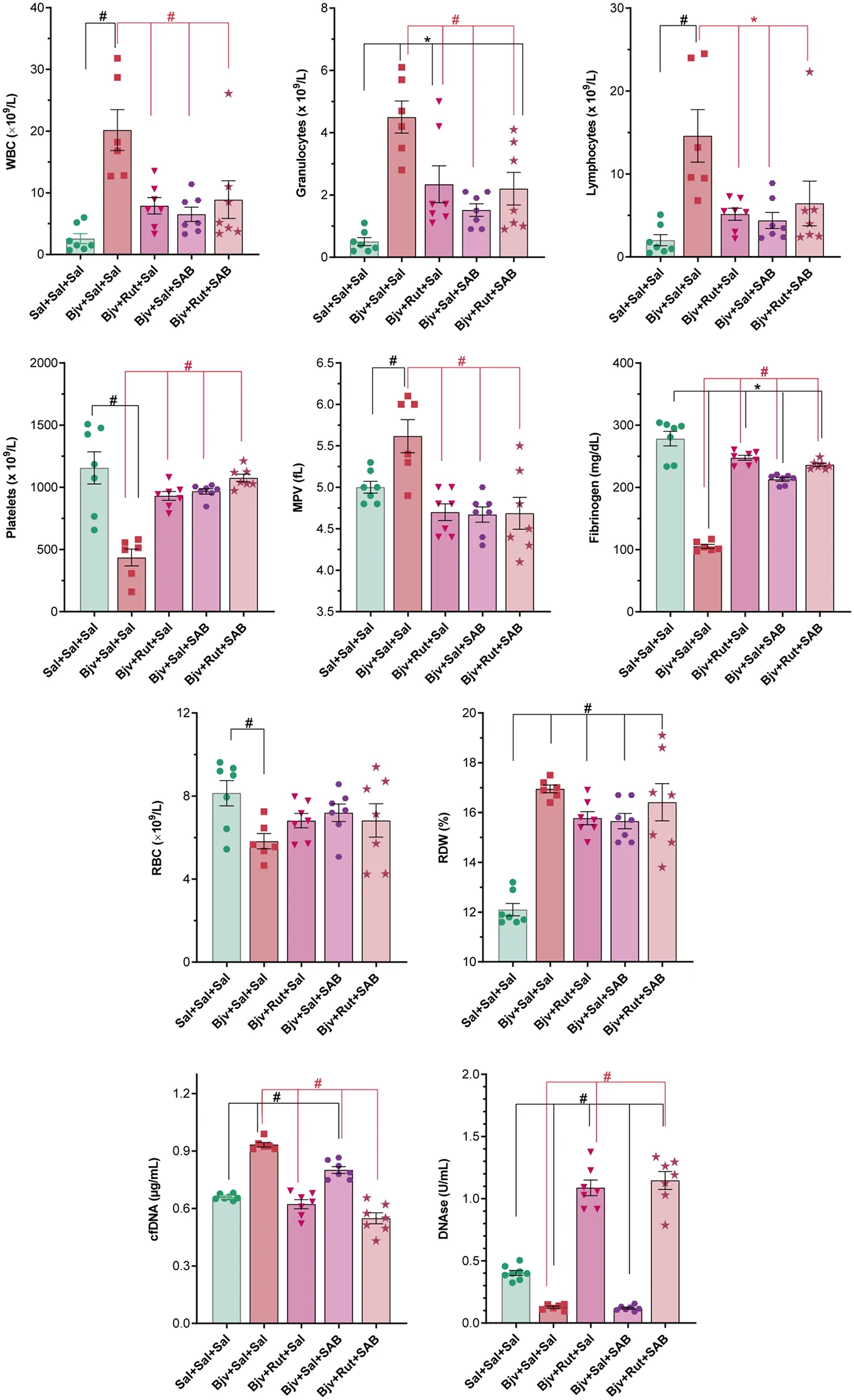
Levels of inflammatory, hematological, and hemostatic markers in mice 72h after i.d. injection of a high dose of Bjv, and treated with saline, nano-rutin (p.o), SAB (i.v.) or nano-rutin+SAB (see Fig 2). One-way ANOVA test was used, followed by the Bonferroni *post hoc* test. * p < 0.05, # p < 0.001 when compared to the negative control group (Sal + Sal + Sal). (black symbols and lines); * p < 0.05, # p < 0.001 when compared to the positive control group (Bjv + Sal + Sal) (red symbols and lines). Data are expressed as mean ± s.e.m. (n = 7/group).

cfDNA levels peaked in the Bjv + Sal + Sal group, whereas nano-rutin-treated groups exhibited lower levels, comparable to or below control values (Sal + Sal + Sal; Fig 9i). Conversely, SAB alone (Bjv + Sal + SAB) provided lower protection against cfDNA elevation. Altogether, these findings indicate that nano-rutin effectively prevents the rise in circulating cfDNA levels. On the other hand, plasma DNase activity was reduced in the Bjv + Sal + Sal group, but increased in nano-rutin-treated animals (Fig 9j). In contrast, SAB alone (Bjv + Sal + SAB) failed to restore DNase activity. Importantly, DNase activity showed a significant inverse correlation with cfDNA levels (r = -0.7942, n = 36, p < 0.001). Altogether, these findings link higher DNase activity to reduced circulating cfDNA levels, although the present study could not establish a causal relationship between these variables.

In recent years, cell-free DNA (cfDNA) has emerged as an important link between innate immunity, inflammation and coagulation [67,68]. Given the close interplay among these processes in *B. jararaca* snakebite envenomation, cfDNA levels were measured in plasma samples from mice evaluated for dermonecrosis to determine whether cfDNA could serve as a systemic marker for the early diagnosis, progression and severity of Bjv-induced necrosis. In addition, DNAse activity was assessed in plasma samples to investigate its potential involvement in the pathogenesis of dermonecrosis and in tissue recovery. cfDNA consists of extracellular DNA fragments present in the circulation, which are released by all cells, through multiple mechanisms, including apoptosis, necrosis, and NETosis (the release of Neutrophil Extracellular Traps, NETs), and can be detected in plasma or serum [69,70]. In healthy individuals, cfDNA originates predominantly from hematopoietic cells (platelet formation by megakaryocytes, erythroblast enucleation, granulocytes and monocyte/macrophages) [71,72]. Under physiological conditions, cfDNA is generally present at low concentration in the bloodstream. However, increased release of cfDNA into the circulation, although nonspecific, may serve as a marker of tissue injury. Elevated cfDNA levels have been reported in various pathologic conditions associated with dysregulated inflammation, cancer, sepsis, psoriasis, burn injuries, and tissue necrosis [67,73–76]. The clearance of cfDNA from the circulation occurs through at least three mechanisms: active uptake by the reticuloendothelial system in the liver and spleen (the primary clearance pathway), passive renal filtration, and direct enzymatic degradation by DNAses [70]. DNAses are endonucleases that hydrolyze double-stranded DNA, contributing to the degradation of chromatin released during necrosis, free extracellular DNA and NETs, thereby exerting anti-inflammatory effects [77]. Two extracellular DNAse1 and DNAse1L3 play a major role in NET degradation [78,79]. DNAse1 is highly expressed in parotid glands, kidneys and small intestine in mice [79,80], and is also secreted by macrophages [81]. DNAse1L3 expression occurs in myeloid cells and is induced by IL-4 [82]. Following extracellular DNA degradation by extracellular DNAses, cfDNA is formed and can be detected in plasma.

In our experimental protocol, cfDNA levels varied significantly among the experimental groups (Fig 9i). Compared with the negative control group (Sal + Sal + Sal), the highest cfDNA levels were noted in the Bjv + Sal + Sal group 72 h after i.d. Bjv injection, indicating extensive cfDNA release as a consequence of severe tissue damage and the inflammatory response associated with necrotic lesion formation. In contrast, the groups treated with nano-rutin (Bjv + Rut + Sal or Bjv + Rut + SAB) exhibited lower cfDNA levels compared with the Bjv + Sal + Sal group, indicating that nano-rutin effectively attenuated cellular damage and the systemic release of cfDNA. The Bjv + Sal + SAB also showed reduced cfDNA levels compared with the Bjv + Sal + Sal, but this reduction was less pronounced than that observed in the groups treated with nano-rutin (Fig 9i). Regression analysis of necrosis area (Fig 8b) versus cfDNA levels showed a strong positive correlation (r = 0.788, n = 28, p < 0.001), suggesting that circulating cfDNA may reflect the extent of tissue injury in this experimental model. Moreover, these results are consistent with a previous report [83] associating increased cfDNA levels with venom-induced tissue damage in human patients, and showing that the effective control of inflammation leads to a significant reduction of cfDNA levels. Taken together, these results highlight the protective role of nano-rutin in limiting cfDNA release following envenomation, and consequently, enhancing the therapeutic efficacy of SAB.

Plasma DNAse activity was significantly higher in the nano-rutin-treated groups (Fig 9j). There was a tendency for the DNAse activity to be inversely proportional to cfDNA levels, supported by a statistically significant negative correlation (r = -0.7942, n = 36, p < 0.001). The inverse association between plasma DNase activity and cfDNA levels is consistent with a potential role for extracellular DNases in cfDNA clearance. However, because DNase activity and cfDNA were measured at a single time point in this experiment, this association should not be interpreted as evidence of a causal relationship. Neutrophils represent the first line of defense of the innate immune system and are rapidly recruited to injured tissues, where they may persist for several days. During *Bothrops* snakebite envenomation, neutrophils infiltrate and accumulate at the bite site, releasing their nuclear and granular contents [64,84]. NETs are involved in antimicrobial functions and in sterile inflammation, as substances secreted by neutrophils, together with DNA, attract additional immune cells [85]. However, excessive or prolonged NET formation can be detrimental due to their cytotoxic, pro-inflammatory, and prothrombotic properties. Indeed, NETs have been shown to contribute to the pathology of several inflammatory conditions, including tissue injuries induced by snake venoms, owing both to the intrinsic prothrombotic activity and their ability to entrap toxins from *Echis carinatus* and *Naja naja venoms*, resulting in vascular thrombosis and subsequent tissue necrosis [86–89]. *In vitro*, neutrophils incubated with *Bothrops bilineata* venom have been shown to release NETs [90]. Extracellular DNASE1L3 and DNASE1 also digest NETs. [70]. As a limitation, although NETs were not directly identified in histological sections herein, their involvement cannot be excluded. Given the observed tendency toward the increase in DNAse activity increase in mice treated with nano-rutin, we propose that it may stimulate DNAse release, thereby facilitating extracellular DNA clearance and contributing to improved wound healing.

Collectively, data from necrosis area, cfDNA levels, and histopathological analyses demonstrate that nano-rutin administration – particularly when combined with antivenom (SAB) – reduces the severity of local tissue damage induced by Bjv. In contrast, SAB alone, as reported in the literature [14], was insufficient to prevent the development of Bjv-induced local and necrotic effects. Nonetheless, the marked reduction in necrotic area observed in the group treated with nano-rutin and SAB underscores the need for additional studies to elucidate the molecular mechanisms underlying this interaction so that the observed protective effect of rutin may be well explained.

It is important to highlight that the compounds used as vehicle to dissolve rutin and facilitate its rapid absorption by the gastrointestinal tract are generally recognized as safe (GRAS) for human use and widely utilized in the pharmaceutical industry to enhance the bioavailability of hydrophobic compounds, such as rutin. Glycerin triacetate and 1,2-propylene glycol are standard, low-toxicity solvents metabolized through natural pathways, while kolliphor RH 40 and labrasol act as non-ionic surfactants known for their high gastrointestinal tolerance and ability to improve drug permeation without significant mucosal irritation [91]. Clinical and toxicological data indicate that these components are safe at the concentrations typically used in oral and topical delivery systems, provided the total daily intake remains within established pharmacological limits [92].

Several limitations should be considered when interpreting the present findings. First, the experimental design evaluated selected early time points (6 and 12 h) for determining most analytes, and a 72-h endpoint for dermonecrosis; therefore, our results do not provide information on longer-term outcomes or wound healing beyond this timeframe. Second, the potential effects of nano-rutin on envenomation-induced renal injury remain unknown, requiring further experiments to address this issue. Third, although increased DNase activity was associated with lower cfDNA levels, NETs were not directly identified or quantified, and a causal relationship between DNase activity, cfDNA clearance, and tissue recovery cannot be established. Fourth, the study did not directly compare the effects of SAB administered 10 min after envenomation with the delayed 1h administration used in our experimental protocol. Such studies would be useful to determine the extent to which treatment timing contributes to the observed differences between nano-rutin and SAB. Finally, this study was performed in a murine model of snakebite envenomation, and therefore the findings cannot be directly extrapolated to a clinical setting without further clinical trials.

Taken together, the findings presented herein support the hypothesis that the use of nano-rutin alongside SAB may represent a promising strategy for controlling the local and systemic effects of envenomation, which remain major clinical challenges in the treatment of *Bothrops* snakebites, particularly when SAB administration is delayed. These results reinforce the potential of the combination of nano-rutin and SAB to complement the therapeutic properties of each other, reducing both systemic toxicity and local inflammatory stress. We believe that nano-rutin may be a drug that represents an important advance in the clinical management of snakebites.

## Conclusions

In conclusion, nano-rutin administered after Bjv exposure showed protective effects against various local and systemic manifestations of experimental envenomation, including hemorrhage, dermonecrosis, inflammatory alterations, and selected hemostatic disturbances. SAB administration improved several systemic parameters, while the combined sequential administration of nano-rutin and SAB generally produced greater protection than either treatment alone for several outcomes. These findings support further investigation of nano-rutin as an adjunct therapeutic approach, particularly for limiting local tissue damage during the interval between snakebite and access to antivenom, particularly in geographically remote areas.

## Supporting information

S1 Fig Counts of circulating monocytes in mice 6 and 12 after administration of Bjv or saline, and treatment with nano-rutin (p.o., 10 min after Bjv administration), SAB (i.v. 1 h after Bjv administration) or both. Two-way ANOVA test was used, followed by the Bonferroni post hoc test. # p < 0.001 when compared to the negative control group (Sal + Sal + Sal) (black symbols and lines). Data are expressed as mean ± s.e.m. (n = 10/group).
(PDF)

S1 Table Spreadsheet containing individual values of mice used in experiments. (XLSX)
