# Supplementary material for: Rutin (quercetin-3-rutinoside) administered orally after experimental Bothrops jararaca envenomation prevents hemostatic, inflammatory and hemorrhagic disturbances
Source: PLoS Negl Trop Dis. 2026 Oct 6;20(10):e0014786. doi: 10.1371/journal.pntd.0014786 (PMC13657293; doi:10.1371/journal.pntd.0014786)
Supplement: Supplementary file 1 [file pntd.0014786.s001.pdf]

Supplementary figure 1

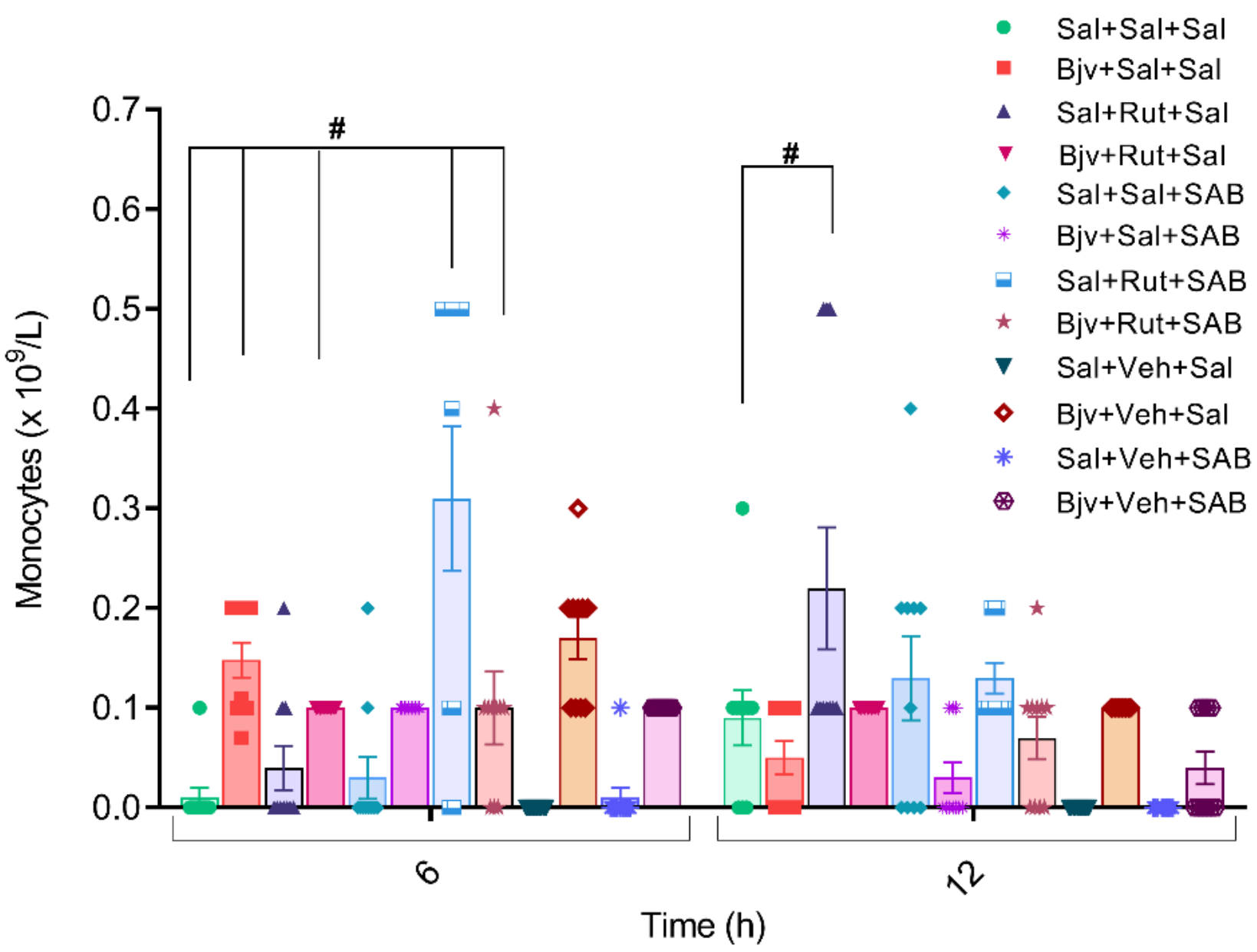

Supplementary Figure. Counts of circulating monocytes in mice 6 and 12 after administration of Bjv or saline, and treatment with nano-rutin (p.o., 10 min after Bjv administration), SAB (i.v. 1 h after Bjv administration) or both. Two-way ANOVA test was used, followed by the Bonferroni *post hoc* test. # p<0.001 when compared to the negative control group (Sal+Sal+Sal) (black symbols and lines). Data are expressed as mean ± s.e.m. (n=10/group).
